# Fluid Lavage of Open Wounds (FLOW): design and rationale for a large, multicenter collaborative 2 × 3 factorial trial of irrigating pressures and solutions in patients with open fractures

**DOI:** 10.1186/1471-2474-11-85

**Published:** 2010-05-06

**Authors:** Flow Investigators

**Affiliations:** 1 McMaster University, 293 Wellington Street North, Suite 110, Hamilton, Ontario L8L 8E7, Canada

## Abstract

**Background:**

Open fractures frequently result in serious complications for patients, including infections, wound healing problems, and failure of fracture healing, many of which necessitate subsequent operations. One of the most important steps in the initial management of open fractures is a thorough wound irrigation and debridement to remove any contaminants. There is, however, currently no consensus regarding the optimal approach to irrigating open fracture wounds during the initial operative procedure. The selection of both the type of irrigating fluid and the pressure of fluid delivery remain controversial. The primary objective of this study is to investigate the effects of irrigation solutions (soap vs. normal saline) and pressure (low vs. high; gravity flow vs. high; low vs. gravity flow) on re-operation within one year among patients with open fractures.

**Methods/Design:**

The FLOW study is a multi-center, randomized controlled trial using a 2 × 3 factorial design. Surgeons at clinical sites in North America, Europe, Australia, and Asia will recruit 2 280 patients who will be centrally randomized into one of the 6 treatment arms (soap + low pressure; soap + gravity flow pressure; soap + high pressure; saline + low pressure; saline + gravity flow pressure; saline + high pressure). The primary outcome of the study is re-operation to promote wound or bone healing, or to treat an infection. This composite endpoint of re-operation includes a narrow spectrum of patient-important procedures: irrigation and debridement for infected wound, revision and closure for wound dehiscence, wound coverage procedures for infected or necrotic wound, bone grafts or implant exchange procedures for established nonunion in patients with postoperative fracture gaps less than 1 cm, intramedullary nail dynamizations in the operating room, and fasciotomies for compartment syndrome. Patients, outcome adjudicators, and data analysts will be blinded. We will compare rates of re-operation at 12 months across soap vs. saline, low pressure vs. high pressure, gravity flow pressure vs. high pressure, and low pressure vs. gravity flow pressure. We will measure function and quality of life with the Short Form-12 (SF-12) and the EuroQol-5 Dimensions (EQ-5D) at baseline, 2 weeks, 6 weeks, 3 months, 6 months, 9 months, and 12 months after initial surgical management, and measure patients' illness beliefs with the Somatic Pre-Occupation and Coping (SPOC) questionnaire at 1 and 6 weeks. We will also compare non-operatively managed infections, wound healing, and fracture healing problems at 12 months after initial surgery.

**Discussion:**

This study represents a major international effort to identify a simple and easily applicable strategy for emergency wound management. The importance of the question and the potential to identify a low cost treatment strategy argues strongly for global participation, especially in low and middle income countries such as India and China where disability from traumatic injuries is substantial.

**Trial Registration:**

This trial is registered at ClinicalTrials.gov (NCT00788398).

## Background

Orthopaedic injuries represent 67% of injury admissions to Canadian hospitals [1]. It is estimated that by 2020, disability from traffic accidents (the major cause of fractures) will rank in the top 3 of all causes of disability [2].

Orthopaedic injuries are even more common internationally. Accelerated urbanization and industrialization in India and China, which represent 40% of the world's population, have resulted in an alarming increase in traumatic injuries. A vehicular accident is reported every three minutes and a death every 10 minutes on Indian roads. For every death, 3 patients survive and live with disability [3].

Open fractures account for an estimated 250,000 fractures in North America annually [4]. These open fractures are often complicated by infections, wound healing problems, and failure of fracture healing-many of which necessitate a re-operation. Open fractures are designated as surgical emergencies and require urgent treatment.

Infections can occur in up to 50% of open fractures that are severe or become grossly contaminated due to the mechanism of their injury [5,6]. Infection can lead to both wound and fracture healing delays [7]. The additional treatment required to manage infections, as well as wound and bone healing complications, leads to increased health care costs, and additional adverse impact on patients' quality of life.

Current management of grossly contaminated fractures includes careful handling of the damaged soft tissues, as well as stabilization of the bone [8,9]. The single most important surgical step in the initial management of open fractures is a thorough irrigation and debridement [4,9,10]. Removal of all contaminated tissue and foreign matter is necessary to prevent infection, support wound healing, and promote fracture healing. Surgeons accomplish debridement with careful removal of all visible debris and necrotic tissue along with copious irrigation of the wound. There is, however, currently no consensus regarding the optimal approach to irrigating the wounds during the initial operative procedure. Multiple options exist for irrigation solutions and the delivery of fluids.

### Experimental Studies Evaluating the Effect of High and Low Pressure Wound Irrigation

Advocates of high-pressure irrigation believe that higher pressures optimally remove all particulate matter and contamination [11-16]. Low pressure advocates believe that low pressure irrigation may damage bone to a lesser extent than high-pressure irrigation, thus preserving bone architecture [14,17-22].

We have conducted a series of laboratory investigations using in-vitro models of contaminated tibial shaft fracture, rat models of fracture healing, and cell culture models of bone nodule formation. Our experimental data suggests high pressure lavage may be more effective than low pressure lavage for removing debris and bacteria from contaminated open wounds after a 3 hour delay [5,20,23]. However, this efficacy in removing debris and bacteria comes at the expense of damage to the bone tissue [18,20], bacterial propagation into the intramedullary canal of the fractured bones [18], and promotion of stem cell differentiation away from bone forming cells (osteoblasts) toward the adipocyte cell types [24]. These cellular level effects also translate into a significant reduction in *in-vivo *strength of fracture healing. Mechanical testing of 36 fractured rat femora after 3 weeks of healing revealed a 37% lower peak bending force and stiffness in animals treated with high pressure irrigation compared to the low pressure groups (p < 0.05) [17].

While findings are not always consistent [13,16], the weight of experimental evidence suggests a trade off between greater efficacy in removing particulate matter and bacteria with high pressure irrigation with the disadvantage being the potential for bone damage, driving particulate matter deeper into bone and tissues and delaying bone healing. The lack of compelling clinical evidence suggests the need for a randomized controlled trial of varying irrigating pressures in patients with open fractures.

### Experimental Studies Evaluating the Effect of Various Irrigating Solutions

The type of irrigating solution and its effect on the efficacy of wound debridement remain controversial. Although experimental studies have evaluated several irrigation additives including antiseptics, antibiotics, and surfactants (soap), few have revealed promise beyond the current common standard solution--normal saline.

Experiments suggest antiseptics are toxic to the host cells [4,25-29]. Although some investigators have promoted irrigation with antibiotic solutions (such as bacitracin), concerns about allergic reactions [30], increased cost [31], promotion of antibiotic resistance, and unproven efficacy have limited their use [32]. In an in-vitro study evaluating multiple irrigating solutions, exposure of mouse calvarial cells to 10% ethanol, 10% povidone-iodine, 10% antimicrobial wash, or 4% chlorhexidine gluconate resulted in cell-density decreases of 70%, 63%, 70%, and 69% respectively [5]. Normal saline solution or soap solutions were the only solutions that did not significantly decrease the cell numbers when compared with controls. The antimicrobial wash further led to a significant decline in in-vitro bone formation (bone nodule formed in-vitro) compared to saline solution [5].

Soaps may act as an emulsifier to help cleanse wounds. Soaps are fatty acids with a positively charged tail and a negatively charged carboxylate head. The positively charged hydrocarbon tail is hydrophobic and repels water. The negatively charged carboxylate head is hydrophilic and interacts with water. This unique property allows for oils (dirt) to become suspended by forming micelles which are easily washed away. As the soap-water solution comes in contact with oil/dirt, the hydrophilic carboxylate heads of the soap bond with water molecules, while the hydrophobic hydrocarbon tails are attracted to each other and to the dirt/oils. This leads to the formation of micelles with the hydrophobic hydrocarbon chains and the trapped dirt on the inside and the hydrophobic carboxylate heads on the exterior of the spherical surface. The negative charged carboxylate heads give the micelles (which now have the dirt/oil trapped in the center) a negative charge, which causes them to repel each other and remains dispersed in the water. While suspended, they can be washed away, theoretically cleansing the wound.

We, along with other investigators, have shown in laboratory and animal models that soap solution is more effective in removing bacteria and particular matter from wounds and bone than normal saline [5,10,26,33,34], without toxic effects to soft tissues and bone [5]. We have further shown a possible synergy between soap and low pressure irrigation [5]. The use of a soap solution under low pressure pulsatile irrigation removed the greatest number of bacteria from the contaminated tibia when compared to either the soap alone, or low pressure irrigation with saline alone (p < 0.01) [5].

The potential efficacy of soap solution in removing particulate matter, oil and bacteria from contaminated open wounds requires confirmation in a definitive trial. At pennies per application, soap offers a low cost, globally applicable, simple intervention that may reduce infections, as well as wound and bone healing complications following open fractures.

### Inconclusive Clinical Evidence

Soap solution has been evaluated by a single surgeon in a randomized trial of 400 patients with 458 open fractures [31]. At a mean 1.3 year follow up, soap solution (80 mL per 3L bag of normal saline) demonstrated a trend towards a decreased risk of infection compared to an antibiotic solution (100,000 U of bacitracin per 3L normal saline) (13% vs. 18%, relative risk 0.74, 95% confidence interval 0.45-1.26, p = 0.2). The study reported a significant reduction in wound healing complications with soap compared to antibiotic (4% vs. 9.5%; p = 0.03). While this study provides some support for the efficacy of soap solution, its findings are limited by relatively small sample size, lack of generalizability to other centers or countries, unconvincingly concealed randomization, and unblinded non-independent adjudication of the primary outcome.

A recent randomized controlled trial of 21 patients with traumatic open wounds [15] compared two alternative high pressure irrigating devices, one delivering 40 p.s.i. and the other delivering above 5,000 p.s.i. pressure to the wound. The investigators reported a similar efficacy in both high pressure devices. This very small study provides limited data suggesting that irrigation pressures of 40 p.s.i. or greater provide similar efficacy to higher pressures; the relative effect of lower pressure irrigation (less than 40 p.s.i.) remains unaddressed.

### Multinational Survey: Uncertainty Regarding Optimal Management, and Support for a Large Trial

We have conducted two surveys [24,35] to explore surgeons' views regarding wound irrigation. Of 577 orthopaedic surgeons managing open tibial fractures who responded to our first survey, 39% preferred high and 45% low-pressure irrigation in their treatment of open wounds [24].

We mailed our second survey to members of the Canadian Orthopaedic Association and delivered it to attendees of an international fracture course (AO, Davos, Switzerland) [35]. Of the 1,764 surgeons who received the questionnaire, 984 (55.8%) responded. In the management of open wounds, 676 (70.5%) favoured normal saline alone. Only 12 surgeons (1.3%) routinely used a soap solution. Although the majority of surgeons [695 (71%)] preferred what they called "low pressure" when delivering the irrigating solution to the wound, there was considerable variation in what pressures constituted high versus low pressure lavage. Based upon the definitions provided, the majority (63.7%) were actually delivering what would constitute "high" irrigating pressures to the wound. In summary, current practice reflects the use of normal saline and higher irrigating pressures [35].

Of the respondents, 803 (84.8%) supported a clinical trial evaluating outcomes following the use of different irrigating solutions and 730 (77.6%) supported a trial of irrigating pressures. Most surgeons [889 (94.2%)] reported they would change their practice if a large RCT showed a clear benefit of an irrigating solution. The majority of surgeons [765 (80.6%)] believed that a particular irrigating solution would need to reduce the risk of infection compared to a standard by at least 25% to change practice. As a final confirmation of support, 612 surgeons reported they would participate in a randomized trial to resolve the controversy [35].

### Pilot Randomized Trial

We have successfully completed a pilot RCT using a factorial design to assess the feasibility of the proposed definitive FLOW trial. One hundred and eleven patients with open extremity fractures were randomized in permuted blocks using a customized web-based/telephone randomization system, to receive either soap or saline solution and either low or high pressure irrigation. Patients, outcome assessors and data analysts were blinded to treatment allocation. The primary outcomes of the pilot study were the rates of reoperation to treatment infection, non-union, and would healing problems in open fracture wounds. Our pilot study demonstrates: 1) our ability to recruit patients for the definitive trial; 2) investigators' compliance with key aspects of the protocol; 3) maintenance of data quality; 4) maintenance of high follow up rates; 5) our ability to organize trial procedures (randomization, data management) in a multinational trial; 6) The study results are consistent with a possible benefit of soap and low pressure irrigation. We have also used the pilot to develop and revise Case Report Forms (CRFs) and the Manual of Operations for Investigational Sites for the pivotal FLOW trial.

### Study Objectives

The objectives of this study are to determine the effects of irrigation solutions (soap vs. normal saline) and irrigation pressures (gravity flow vs. high; low vs. high; gravity flow vs. low) on open fractures of extremities. The primary objective is to determine, in patients operatively treated for open fractures of the extremity, the effects of irrigation solutions (soap vs. normal saline) and irrigation pressures (high vs. low; high vs. gravity flow; low vs. gravity flow) on re-operations within one year after initial surgery. The secondary objective is to investigate the impact of irrigation solution (soap vs. normal saline), pressure (high vs. low; high vs. gravity flow; low vs. gravity flow), and illness beliefs on patient function and quality of life at one year. We will also compare non-operatively managed infections, wound healing, and fracture healing problems within 12 months after initial surgery.

## Methods and Design

### Overview of Study Design

This study is a multi-center, blinded, randomized controlled trial, using a 2 × 3 factorial design. Patients are randomized to one of 6 treatment arms: 1) castile soap solution and low pressure, 2) castile soap solution and high pressure, 3) castile soap solution and gravity flow pressure, 4) saline solution and low pressure, 5) saline solution and high pressure, and 6) saline solution and gravity flow pressure (Table 1). We have chosen a factorial design to optimize the efficiency and reduce overall trial cost [36,37]. We will be able to efficiently and simultaneously investigate two interventions (irrigating pressure and irrigating solution) by including all participants in both analyses. For instance, when analyzing the effect of soap solution versus saline, we will include patients across different pressure groups (including gravity flow, low pressure, and high pressure); in the comparison of high versus low pressure, we will include patients using soap solution and saline. The United States Department of Defense-OETRP grants review committee urged us to include a very low pressure group (gravity flow irrigation) arm to maximize the applicability of the trial, and we have done so. The Research Ethics Board at each clinical site has approved the protocol.

**Table 1:** 2 × 3 Factorial Design with a Total of 2280 Patients and 380 Patients per Arm

	Gravity Flow Pressure	Low Pressure	High Pressure	Total
**Soap solution**	380	380	380	1140

**Saline**	380	380	380	1140

Total	760	760	760	2280

### Randomization

We will randomize patients using variable block sizes to ensure effective stratification. An automated internet-based randomization system based at the CLARITY Methods Center at McMaster University (available 24 hours/day), which we have used successfully for other multicenter trials, will ensure concealed randomization of eligible consenting patients. To ensure a prognostic balance between key factors, we will stratify patients by center and the type of Gustilo-Anderson open fracture (Types I and Type II versus Type III). We will apply permuted randomization, using block sizes of 6 or 12 at random, to allocate patients.

Once patients or their proxy have provided informed consent and the operating or attending surgeon has evaluated the open fracture wound, the investigator or designated study team member will contact the automated randomization system at the Methods Center. Patients will be randomized to one of 6 treatment groups: 1) castile soap solution and low pressure, 2) castile soap solution and high pressure, 3) castile soap solution and gravity flow pressure, 4) saline solution and low pressure, 5) saline solution and high pressure, and 6) saline solution & gravity flow pressure.

### Patient Selection

Patients who meet the eligibility criteria outlined below are to be included in the FLOW study. Only one fracture per patient is to be included. For patients with multiple eligible open fractures, the study fracture will be the most severe (i.e. the greatest Gustilo-Anderson Type).

#### Eligibility Criteria

Eligible patients will meet all the following inclusion criteria: 1) men or women who are 18 years of age or older, 2) adequate x-ray confirmation of an open fracture, 3) open fractures (Gustilo-Anderson Types I-IIIB) (Table 2), and 4) fracture requiring operative fixation.

**Table 2:** Gustilo-Anderson Classification of Open Fractures

Open fracture type	Characteristics
Type I	Clean wound smaller than 1 cm in diameter, simple fracture pattern, no skin crushing.

Type II	A laceration larger than 1 cm but without significant soft tissue crushing, including no flaps, degloving, or contusion. Fracture pattern may be more complex.

Type III	An open segmental fracture or a single fracture with extensive soft tissue injury. Also included are injuries older than 8 hours. Type III injuries are subdivided into three types.

Type IIIA	Adequate soft tissue coverage of the fracture despite high energy trauma or extensive laceration or skin flaps.

Type IIIB	Inadequate soft tissue coverage with periosteal stripping. Soft tissue reconstruction is usually necessary.

Type IIIC	Any open fracture that is associated with an arterial injury that requires repair.

We will exclude patients meeting any of the following criteria: 1) open fractures with an associated vascular deficit (Gustilo-Anderson type IIIc), 2) known allergy to detergents or castile soap ingredients, 3) previous wound infection or history of osteomyelitis in the injured extremity, 4) previous fracture with retained hardware in injured extremity that will interfere with new implant fixation, 5) surgical delay to operative wound management greater than 24 hours from hospital admission, 6) use of immunosuppressive medication within 6 months, 7) immunological deficient disease conditions (e.g. HIV), 8) fracture of the hand (metacarpals and phalanges), 9) fracture of the toes (phalanges), 10) likely problems, in the judgment of the investigators, with maintaining follow-up, 11) previous randomization in this study or a competing study, 12) patient is a prisoner or is at high risk of incarceration during the follow-up period (clinical sites located outside of the United States may enroll prisoners or those at high risk of incarceration with the approval of their local IRB/REB), and 13) failure to obtain informed consent.

#### Patient Recruitment and Screening

**Figure 1:**
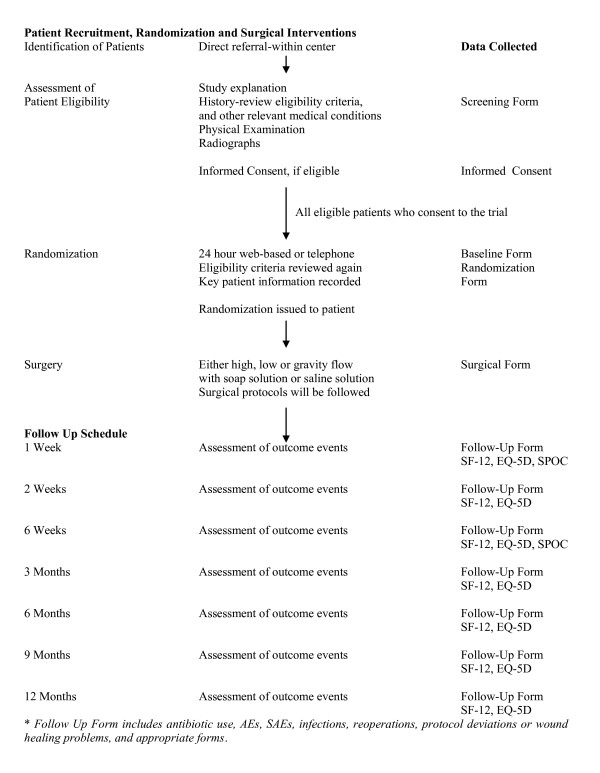
**Trial Conduct Procedure**.

Figure 1 shows the patient identification and screening procedures. Informed consent will be obtained from all eligible patients. If a patient is deemed unable to consent due to being temporarily incapacitated (i.e. due to trauma, pharmacological or other influence), informed consent may be obtained from the patient's legally authorized representative. When the patient is deemed no longer incapacitated, the patient will be approached regarding the study and informed consent will be obtained from the patient for ongoing participation in the study. If the patient declines continued participation, the patient will be withdrawn from the study.

We will register all patients who meet the eligibility criteria and document reasons for failure to randomize. We will document all patients screened for eligibility and record patients as: 1) eligible and included, 2) eligible and missed, and 3) excluded. The blinded Central Adjudication Committee (CAC) will adjudicate all situations where eligibility is in doubt.

### Study Interventions

#### Irrigating Solutions

Patients will be randomized to have their open fracture wound irrigated with either soap or normal saline. In the operating room, surgeons will use sterile technique to inject 80 mL of the clear liquid soap (Castile Soap, Triad Medical Inc. Franklin, Wisconsin - 17% concentration in de-ionized water preserved in 90 mL bottles) with a sterile syringe into a 3L bag of normal saline. Our choice of castile soap and dosing is based upon experimental evidence, a recent clinical trial that used this formulation without adverse effects [31], and our pilot study that confirmed its safety. Patients randomized to the normal saline group (control) will receive sterile normal saline provided in 3L bags.

We will standardize the minimum amount of soap or saline solution based upon the severity of open fracture wound according to the Gustilo-Anderson Classification (Type I - 3 Litres, Types II and III - 6 Litres). We based these volumes on our international survey data [35] to reflect current standards and management protocols.

#### Irrigating Pressures

Patients will also be randomized to have the solutions delivered to the open fracture wound by gravity flow (1-2 p.s.i.), low-pressure irrigation (5-10 p.s.i.), or high-pressure irrigation (>20 p.s.i.) with a battery operated irrigator [Stryker Surgilav or Zimmer Pulsavac Plus].

Gravity flow irrigation will be standardized across participating centers as 3L bags of normal saline (alone or with soap solution) suspended 6-8 feet above floor level (2-5 feet above the table) using an I.V. pole. Irrigation tubing (measuring 1/4 - 3/8 inch inner diameter) will be connected to the 3L bag and secured with a stopcock (or compressive device) until ready for use. At the time of irrigation, the stopcock (or compression device) will be released and gravity flow irrigation of the open wound will occur. A large basin collecting the runoff will be suctioned by standard intraoperative suction tubing. No pressure will be applied to the bag of solution.

To ensure standard low and high pressure delivery, we will require the irrigator to be one of two devices [Stryker Surgilav or Zimmer Pulsavac Plus] at all participating sites. For low pressure delivery, the high flow trauma tip of the Stryker Surgilav (at the low pressure setting) or the shower tip of the Zimmer Pulsavac Plus (at the low pressure setting) will be used to deliver a pressure of 6 p.s.i or 5.8 p.s.i, respectively. For the high pressure delivery, the multi-orifice tip of the Stryker Surgilav (at the high pressure setting) or the shower tip of the Zimmer Pulsavac Plus (at the high pressure setting) will be used to deliver a pressure of 30 p.s.i or 23 p.s.i., respectively. The irrigator tip will be held perpendicular to and 5 cm above the wound.

#### Standardization of Procedures and Peri-Operative Care

We will standardize key aspects of peri-operative care and technical aspects of the initial irrigation and debridement procedure.

#### Antibiotics

Pre-operative I.V. antibiotics will be administered commencing on diagnosis. Postoperative, I.V. antibiotics will be administered for at least 24 hours post-surgery. Specific antibiotics will be used at the discretion of the attending surgeon. The recommended guidelines include: Cephalosporin (Ancef) I.V. for Grade I-II injuries, Cephalosporin (Ancef) I.V. and Aminoglycoside (Gentamycin) I.V. for Grade III injuries, and Cephalosporin (Ancef) I.V., Aminoglycoside (Gentamycin I.V) and penicillin for gross contaminated injuries. For large open wounds (Types III), temporary local antibiotic administration will be permitted (bead pouch) until definitive wound closure. All antibiotics that are prescribed for the randomized fracture are to be recorded on the CRFs.

#### Wound Management

Prior to randomization, we will record whether the attending surgeon plans to use antibiotic beads or antibiotic osteobiologics and if the attending surgeon plans to use negative pressure wound therapy (wound vacs) to treat the patient's randomized open fracture wound. The FLOW randomization system will capture this information prior to the treatment allocation being provided. Since the attending surgeons will not be blinded to the treatment allocation and bias may be introduced, we strongly encourage surgeons to use antibiotic beads or antibiotic osteobiologics, and negative pressure wound therapy (wound vacs) only if they indicated this prior to randomization. We will record the actual use of antibiotic beads or antibiotic osteobiologics and negative pressure wound therapy (wound vacs) on the case report forms and we will document any discrepancies with the initial plan.

Intra-operatively, surgeons will prepare and drape the injured extremity using sterile technique. Iodine-based or chlorhexidine-based initial wound scrubs will be allowed for extremity preparation. Surgeons will initially remove all gross debris, contaminants, and dead tissue (muscle, fat, fascia, skin, or bone). Adequacy of the debridement will be judged by colour, consistency, contractility, and bleeding of the muscle as well as complete eradication of contaminated and necrotic tissue including nonarticular devitalized bone. Delayed wound closure, split thickness skin grafting, or muscle flaps should occur by 7-14 days following the initial surgery when possible. Surgeons will repeat the irrigation and debridement procedure until the open wound is clean and soft tissues viable. Patients will receive the same irrigating pressure and solution to which they were initially randomized for all subsequent irrigations and debridements.

#### Fracture Stabilization

Fracture stabilization will be at the surgeon's discretion. Surgeons should stabilize the fractures using current best practices. These include the following guidelines: 1) definitive fixation should be in place by 14 days from the initial operative wound irrigation and debridement as soft tissue allows, 2) temporizing fracture stabilization (external fixation) for grossly contaminated (Type II or Type III) wounds if used should be spanning external fixation outside the zone of the injury, 3) definitive fixation for shaft fractures of the lower extremity will include statically locked intramedullary nails (unless very proximal or very distal) [23], and 4) upper extremity fractures should be treated when possible with plates and screws [38]. Due to the varying nature of these traumatic fractures, each fracture should be stabilized as the treating surgeon sees fit. To ensure both feasibility and generalizability, we will not standardize the implants.

### Study Endpoints

#### Primary Study Endpoints

The primary study endpoint is re-operation within 12 months post initial surgery to treat an infection, manage a wound healing problem, or promote fracture healing. Re-operation is defined as a surgery that occurs subsequent to the initial procedure. This composite endpoint of re-operation will include a narrow spectrum of patient-important procedures: irrigation and debridement for infection, revision and closure for wound dehiscence, wound coverage procedures for infected or necrotic wounds, bone grafts or implant exchange procedures for established nonunion in patients with postoperative fracture gaps less than 1 cm, intramedullary nail dynamizations in the operating room, and fasciotomies for compartment syndrome.

Infections will be classified according to the Center for Disease Control (CDC) Criteria [39]. We will define infection in patients as a constellation of clinical symptoms and laboratory examinations. These will include (but are not limited to) fever, erythema/cellulitis, positive tissue cultures, and frank purulent drainage.

Our definition for wound healing problems will follow previously published criteria [31]. Any re-operations related to problems with primary wound healing will be documented. These include: 1) a dehiscence of a suture line, death of a flap or graft, or failure to heal which is not due to underlying deep infection (drainage of purulent fluid and positive cultures) or 2) problems with secondary healing that include failure of the wound to progress to satisfactory closure (wound becomes larger over time, failed granulation, or development of necrosis all requiring further intervention).

Diagnosis of nonunion will include a failure of the fracture to progress towards healing as observed by the treating surgeon and that requires further intervention to promote healing either surgical (i.e. bone graft) or non-surgical (i.e. bone stimulator). Final consensus on nonunion will be determined by the Central Adjudication Committee.

The following conditions are not considered outcome events: 1) planned secondary interventions from initial surgical procedures and 2) any re-operations to promote fracture healing in patients with post-operative fracture gaps greater than 1 cm.

#### Secondary Study Endpoints

The secondary study endpoints include patient function and quality of life measured by the Short Form-12 (SF-12) and the EuroQol-5 Dimensions (EQ-5D), non-operatively managed infections, wound healing problems, and fracture healing problems within 12 months.

#### Adjudication of Study Events

The Central Adjudication Committee, blinded to treatment allocation, will adjudicate re-operations to treat infection, wound healing problems, or fracture healing problems (delayed unions and nonunions), soft tissue procedures without infection in patients who have undergone more than 3 re-operations, and non-operatively managed infections, wound healing problems, and fracture healing problems. The adjudicators may review the patient's initial hospital notes, clinic notes, and x-rays.

### Study Follow-Up

Figure 1 shows the study follow-up timeline. At each follow up, patient conditions and outcomes will be recorded. At 1 and 6 weeks, patients will also complete the the Somatic Pre-Occupation and Coping (SPOC) questionnaire. The SPOC questionnaire is a validated self-administered, 27-item questionnaire that measures patient's illness beliefs.

Any adverse events, re-operations, infections, wound healing problems, antibiotic use relating to the fracture, and protocol deviations will be recorded. Missed follow up or early withdrawal will also be documented.

We will withdraw patients only if patients withdraw consent for participation. We will document the reasons for patient withdrawal from the trial. For those patients who withdraw from other study activities, we will seek their approval to collect clinical data from their medical and hospital charts, and/or to contact them by telephone to ask about the primary and secondary outcomes.

### Protecting Against Sources of Bias

**Figure 2:**
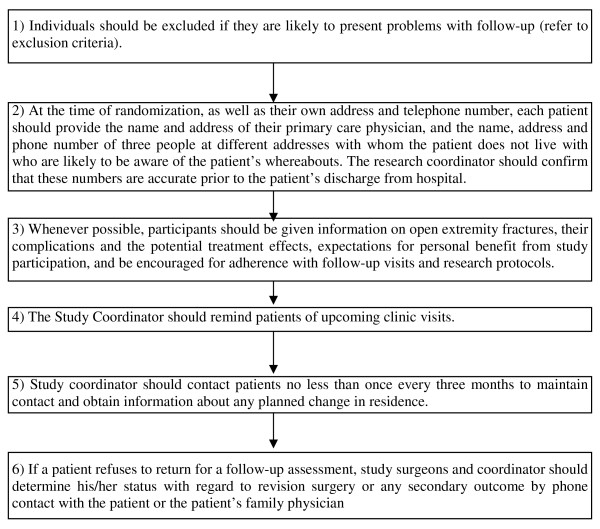
**Strategies to Limit Loss to Follow-Up**.

Patients, outcome adjudicators, and data analysts will be blinded to the study treatment. The operating room team (including the surgeon and study coordinator) cannot be blinded since the equipment they use for the irrigation pressures and the solutions are readily distinguishable. We will implement several procedures to limit loss of follow up (Figure 2). Patients who do not receive the irrigation solution and/or pressure to which they are randomized will be followed as per the study protocol.

### Co-interventions and Contaminations

To minimize the co-interventions, we will standardize the peri-operative procedures and antibiotics use. Patients will receive the surgical management to which they are randomized for the initial irrigation and debridement and for all subsequent irrigation and debridements. To prevent any patients from receiving the wrong solution or pressure, the following three measures will be applied whenever possible: 1) FLOW posters with clear preparation guides will be posted in all emergency operating rooms, 2) soap bottles will be placed in all orthopaedic operating rooms in clearly marked boxes with instructions, and 3) surgeons who complete the subsequent irrigation and debridements will be aware of the patient's treatment allocation. The study coordinator of the clinical site will, if possible, be present during any subsequent irrigation and debridements to ensure that patients receive the treatment to which they are randomized.

### Statistical Plan

#### Sample Size Determination

Our sample size is chosen to identify if there is any difference in effects of pairwise comparisons of the three irrigation pressure groups (high vs. low; high vs. gravity flow; low vs. gravity flow) on re-operations at 12 months (Table 1). This sample size will also allow us to establish if there is a difference between soap and saline (see below). For the comparisons of the three different pressures, we have chosen a two-sided alpha level of 0.05. Given that this applies to three pairwise comparisons, our alpha level for each individual comparison will be, according to Tukey's method, 0.0188 [40].

Our best estimate of the control group re-operation rate is 30%. In a previous randomized trial that involved lower limb open fractures [31], the overall rate of re-operations due to infection, wound healing complications and delayed fracture healing was 46%. In the SPRINT trial, the reoperation rate in 400 patients with open tibial fractures was 27% (95%CI: 22-31). In the FLOW pilot study, which was conducted mainly in North America, the reoperation rate in 111 patients with open fractures was 23% (95% CI 16%-32%). In this pivotal study, we expect that the reoperation rates will be higher in the international sites in India and China. A 25% relative risk reduction associated with one or both of the lower pressures is plausible based on the pilot data. Furthermore, based upon our survey 80% of surgeons will consider a 25% relative difference between treatments important enough to change practice [35]. We anticipate 5% of patients will be lost to follow up given the results of pilot study.

Our experiences in the pilot study, and with centers that have committed to participate in the FLOW definitive study, suggest that we will be able to recruit a total sample size of 2280, 760 per pressure group at the margin of table for a 2 × 3 factorial design (i.e. 380 per cell, Table 1). Table 3 shows our study power for the three pairwise comparisons of alternative pressures given our chosen sample size and given varying control event rates and relative risk reductions. Power is over 80% for relative risk reductions over 28% for control event rate as low as 25%. Power is over 80% for relative risk reduction as low as 24% if our control event rate is as high as 30%.

**Table 3:** The Power of Our Study to Detect the Relative Risk Reduction for Pairwise Comparison of Three Pressure Groups Given Varying Control Event Rate

		Relative risk reduction				
		
		20%	24%	28%	32%	35%
Control event rate	25%	0.49	0.68	**0.84**	**0.93**	**0.97**
	
	27%	0.54	0.73	**0.87**	**0.95**	**0.98**
	
	30%	0.61	**0.80**	**0.92**	**0.98**	**0.99**
	
	33%	0.68	**0.85**	**0.95**	**0.99**	**1.00**
	
	35%	0.72	**0.89**	**0.99**	**1.00**	**1.00**

* Note: We use a two-sided alpha level of 0.0188 for each pairwise comparison of three pressure groups, and the sample size per group at the margin is 760 (380 per cell).

We have the same best estimate of control group re-operation rate (i.e. 30%) for the saline solution effect, based on two randomized trials [31,35]. Given that our pilot data suggested a 37.5% relative risk reduction with soap versus saline, a relative risk reduction of 25% is plausible. For the saline versus soap comparison, we will have a larger number of patients (i.e. 1,140) per group and a higher threshold p-value (0.05 vs. 0.018). Therefore, for any given baseline risk and relative risk reduction, our power will be greater for the saline-soap comparison than for the pressure comparisons.

For our secondary outcomes, we consider an important difference in SF-12 to correspond to a moderate effect as reported by Cohen [41] as well as a minimally important difference in the SF-12 as reported by Ware [42]. In both cases, the value is 1/2 the standard deviation, equivalent to 5 point difference in score. Specifying an alpha level = 0.01, a beta = 0.20 (study power = 0.80), we require a sample of at least 405 patients (135 per pressure group at the margin of the table) to ensure detection of a 1/2 standard deviation improvement.

The EQ-5D correlates well with the Health Utilities Index (HUI) and both have been reported to provide similar estimates of utility [43]. Drummond et al [44] report that 0.03 - 0.04 incremental changes in HUI represent a patient-important difference. For adequate study power, we will need to recruit at least 329 patients per group at the margin of the table (alpha level = 0.01, a beta = 0.20, difference = 0.04, σ = 0.15). Thus, in all circumstances, our desired sample size of 2280 patients (760 per group at the margin of the table) will be sufficient to detect the minimally important differences in our secondary measures of outcome.

### Statistical Methods

#### Primary Analysis

Analyses will include all patients in the groups to which they were randomized. The data analyst and investigators, while conducting the analyses, will be blind to treatment groups. We will use the log-rank test and the Kaplan-Meier survival curve to compare the main effects of irrigating solution (soap vs. saline) and irrigation pressure (high vs. low, high vs. gravity flow, low vs. gravity flow) at the margins of the 2 × 3 factorial design on time to the first re-operation after the initial surgery. We will use a two-sided alpha level of 0.05 for the comparison of irrigation solution and a two-sided alpha level of 0.0188 for pairwise comparison of irrigation solution. We will use Cox model to generate hazard ratio and its associated 95% confidence intervals for each comparison. The analyses will be stratified by center and the type of Gustilo-Anderson open fracture (Types I and II versus Type III).

Adjusted analyses, employing Cox regression, will examine and control for the influence of patient and surgical factors that might be associated with the risk of re-operation, including age, degree of soft tissue injury, upper or lower extremity injury, extent of fracture gap, type of internal fixation, and severity of fracture.

#### Secondary Analyses

We will also examine the interaction of soap with pressure by including the main effects and their interaction terms in the Cox regression with the outcome variable as re-operation. This secondary analysis will have lower power and only large effects will be detectable.

In addition to re-operation, we will also compare the effects of irrigation solution (soap vs. saline) and pressure (low vs. high; gravity flow vs. high; gravity flow vs. low) on the component outcomes, including non-operatively treated fracture healing complications, wound healing problems, and infection (deep and superficial), using log-rank test and Kaplan-Meier survival curve. Adjusted analyses, using the Cox model, will be used to examine and control for the influence of patients and surgical factors.

We will employ the generalized linear model for repeated-measure analysis of variance to examine the effects of time, treatment, and the interaction between the two to compare functional status and quality of life for all comparison groups. We will construct multi-variable regression models to explore the association between SPOC scores and functional outcome at 1-year, as measured by SF-12 physical component summary (PCS) and mental component summary (MCS) scores. We will also examine if SPOC scores at 1 and 6 weeks are similarly predictive.

#### Subgroup Analyses

We plan to conduct two subgroup analyses, both with strong biological rationale and possible interaction effects. The first will compare hazard ratios of re-operation based upon the degree of soft tissue injury (Gustilo-Anderson Type I/II open fractures vs. Gustilo-Anderson Type IIIA/B open fractures). The second will compare hazard ratios of re-operation between fractures of the upper and lower extremity. We will test if the treatment effects differ with fracture types and extremities by putting their main effect and interaction terms in the Cox regression. For the comparison of pressure, we anticipate that the low/gravity flow will be more effective in the Type IIIA-B open fracture than in the Type I/II open fracture, and be more effective in the upper extremity than the lower extremity. For the comparison of solution, we anticipate that soap will do better in the Type IIIA-B open fracture than in the Type I/II open fracture, and better in the upper extremity than the lower extremity.

#### Interim Analysis

We will conduct an interim analysis to monitor the treatment benefits. The interim analysis will be performed when two-thirds of the entire patient follow-up is completed (i.e. 1520 person-years). At this point, 91.7% (1886) patients will have been recruited into the trial.

We use the O'Brien-Fleming Method to calculate the stopping boundary. We will maintain the overall specified type I error rate of 0.05 for the comparison of soap solution versus normal saline, and the threshold 2-sided significance level is 0.012 for the interim analysis. We will maintain the overall specified type I error of 0.0188 for each of the three pairwise comparisons of irrigation solutions, and the threshold 2-sided significance level is 0.003.

The data analyst will present the results of analysis, including confidence intervals, to an independent DMC. No one other than committee members will be aware of the data on which the committee makes its decision, and no one involved in the study will be aware of the content of their deliberations.

#### Ethical Considerations

This study is to be conducted according to US and international standards of Good Clinical Practice (FDA Title 21 part 312 and International Conference on Harmonization guidelines), applicable government regulations and Institutional research policies and procedures. All patients for this study will receive a consent form describing this study and providing sufficient information for patients to make an informed decision about their participation. The consent form has been submitted with the protocol for review and approval by the Research Ethics Board (REB) or Institutional Review Board (IRB) for each clinical study site. The formal consent of a patient, using the REB or IRB-approved consent form, will be obtained before that patient undergoes any study procedure.

## Discussion

Orthopaedic trials have been typically single-center, small studies that often lack sufficient power to reliably evaluate the important treatment effects, and suffer from methodological weakness. Previous orthopaedic trials have also tested only a single surgical intervention per study. The FLOW trial is the first study in orthopaedic traumatic surgery that uses a factorial design to address the treatment effects of two interventions in a single trial. Subsequent to our first large randomized orthopaedic trial (SPRINT) that has set a benchmark of conducting orthopaedic trials [45,46], the FLOW study, which will be the largest trial in orthopaedic surgery, represents our continuing efforts to test surgical interventions and advance research methods in orthopaedic trials.

Our trial will test whether the soap solution and lower irrigation pressure (i.e. gravity flow) reduce the risk of re-operation. These strategies are easily applicable and associated with minimal costs. Establishing their effects will have potentially important clinical and economic implications, in particular to patients in low and middle income countries such as India and China where disability from traumatic injuries is substantial.

The FLOW trial has several important strengths. It will enroll 2280 open fracture patients from clinical sites in North America, Australia, Europe, and Asia - a sample size that ensures sufficient power to reliably detect small but important differences. Our trial uses the factorial design to simultaneously address effects of irrigation solutions and pressures, which improves the efficiency of the evaluation and substantially reduces the trial costs. Our trial has set stringent methodological safeguards against bias. We employ a variable block size with a centralized system to randomize patients; we will blind patients, outcomes adjudicators, and data analysts to treatment allocations; we will standardize peri-operative care and wound management strategies to reduce co-interventions; we will implement strategies to limit the patients lost to follow up; and we will adjudicate the events and address patient eligibility when in doubt through a central adjudication committee.

One major limitation of FLOW is the fact that surgeons cannot be blinded to treatment allocation, leaving the assessment of outcomes and decisions to re-operate vulnerable to bias. To limit such bias, we are using an objective primary outcome and centrally adjudicating all primary outcome events by a group of orthopaedic trauma surgeons blinded to treatment allocation. The primary composite outcome of re-operation provides a patient-important estimate of effect superior to previously described measures such as radiographic fracture healing, delayed unions, and nonunions.

FLOW, as for any trial evaluating two procedures requiring surgical experience, is at risk for differential expertise bias [47]. The procedures for irrigation and debridement, however, are minimally technically challenging. It is unlikely that a substantial difference of expertise is present across operative surgeons. We have standardized peri-operative and wound management procedures that are able to limit the differential use of interventions.

The effects of irrigation strategies on open fractures remain controversial. Prior evidence has suggested potential benefits of soap solution and lower irrigation pressures that have reduced costs. Identifying effective treatment alternatives that reduce both the risk of subsequent operation and health care costs will have an important contribution to orthopaedic practice. This trial will not only potentially change current orthopaedic practice, but also advance research methods for future orthopaedic trials.

## Competing interests

Stryker will provide the Surgilav irrigator, free of charge, to the Chinese sites. Zimmer will provide the Pulsavac irrigator, at a special price, to the sites in North America, Europe, and Australia.

## Pre-publication history

The pre-publication history for this paper can be accessed here:

http://www.biomedcentral.com/1471-2474/11/85/prepub

## FLOW Investigators

The following persons participated in the FLOW Study:

**Study Co-Principal Investigators**: Mohit Bhandari, Gordon Guyatt, and Kyle Jeray; **Steering Committee**: Mohit Bhandari (Chair), Jeff Anglen, Michael Bosse, Gordon Guyatt, Kyle J. Jeray, Susan Liew, Bradley Petrisor, Parag Sancheti, Emil H. Schemitsch, Paul Tornetta, Xin Sun, and Stephen D. Walter; **Central Adjudication Committee**: Mohit Bhandari (Chair), Kyle Jeray, Bradley Petrisor, and Emil H. Schemitsch; **FLOW Methods Centre Staff**: **McMaster University, Hamilton, Ontario, Canada**: Mohit Bhandari, Gordon Guyatt, Bradley Petrisor, Xin Sun, Sheila Sprague, Paula McKay, Diane Heels-Ansdell, Lisa Buckingham, Aravin Duraikannan, and Deborah Maddock; **Greenville Hospital System, Greenville, South Carolina, USA**: Kyle Jeray, Stephanie L. Tanner and Rebecca G. Snider; **Data Monitoring Committee (DMC)**: Doug Altman (Chair), Rajiv Ghandi, and Markus Bischoff; **Site Audit Committee**: Sheila Sprague, Paula McKay, Stephanie Tanner, and Xin Sun; **Medical Monitor for OTRP Funded Sites**: David E. Westberry.

## List of Clinical Sites

USA: Greenville Hospital System, Greenville, South Carolina: Kyle J. Jeray, J. Scott Broderick, David R. Goetz, Michael L. Beckish, Scott E. Porter, Stephanie L. Tanner, Rebecca G. Snider and Shea Bielby; East Texas Medical Centre: Joseph M. Conflitti, Dennis Scott Devinney, and Stephanie Yabrough; University of Missouri Health Care: Gregory J. Della Rocca, Brett D. Crist, Yvonne M. Murtha, Linda K. Anderson and Kelly M. Sullivan; Lahey Clinic Medical Center, Burlington, Massachusetts: Andrew J. Marcantonio, Michael S. Kain, Richard M. Wilk, Richard Iorio, Paul M. Smiley, George Kasparyan, Joshua A. Baumfeld, Eric T. Tolo, Alice A. Hunter, Maximillian Soong, Margaret Lobo, Anthony K. Teebagy, Michael S. Thompson, John F. Tilzey, and John Garfi; Wishard Health Services, Indiana University, Indianapolis, Indiana: Brian H. Mullis, Janos P. Ertl, Jeffrey O. Anglen, J. Andrew Parr, Judd E. Cummings, Ripley Worman, Erin Tobias, Caitlyn Prucinsky, Valda Frizzell and Sara Frizzell; Ohio State University Medical Center, Columbus, Ohio: Laura Phieffer, Ronald Lakatos, Teresa DiMeo, and Skye Macalester; Duke University Medical Center, Durham, North Carolina: Robert Zura, and Maria J. Manson; Miami Valley Hospital, Wright State University, Dayton, Ohio: Michael Prayson, Mike Coffey, and Jason Vourazeris; San Francisco General Hospital, San Francisco, California: Theodore Miclau, Utku Kandemir, Saam Morshed and Tigist Belaye; University of Mississippi Medical Center, Jackson, Mississippi: Matt Graves, Lori Smith, and Zhen Qin; United States Army Institute of Surgical Research and Brooke Army Medical Center, Fort Sam Houston, Texas: Joseph R. Hsu, Joseph C. Wenke, and Donna M. Lopez; Harris Methodist Hospital, Fort Worth, Texas: Cory Collinge, and Tara Weaver; University of California - Irvine, Orange, California: David P. Zamorano, Deanna Lawson, Jeremy Smith, George Whaba, Jason Hofer, and Amy Steinhoff; University of Alabama, Birmingham, Birmhingam, Alabama; Rena Stewart, Holly Waldrop, and Tanya Nix; St. Louis University, St. Louis, MO: Lisa Cannada and Leslie Hill; University of Pittsburgh Medical Center, Pittsburgh, PA: Ivan Tarkin and Arlene Luther; Orthopaedic Associates of Michigan, Grand Rapids, MI: Cliff Jones and Deborah Sietsema; St. Elizabeth Health Center, Youngstown, OH: James Shaer and Barbara Hileman

Canada: Hamilton Health Sciences, Hamilton, Ontario: Bradley Petrisor, Mohit Bhandari, Brian Drew, Dale Williams, Ivan Wong, Krishan Rajaratnam, Desmond Kwok, Rajiv Gandhi, Rick Ogilvie, Tashay Mignott, Laura Quigley, Natalie Kuurstra, Paula McKay, and Sheila Sprague; Ottawa Hospital - Civic Campus, Ottawa, Ontario: Steven Papp, Eugene K. Wai, Allan Liew, Wade Gofton, Vivian Borsella, Julia Foxall, and Liisa Vexler; Queen Elizabeth II Health Sciences Centre: Chad Coles, Ross Leighton, David Amirault, William Oxner, David Alexander, Catherine Coady, Michael Dunbar, Mark Glazebrook, Michael Gross, David Johnston, Gerald Reardon, William Stanish, Kelly Trask, and Gwendolyn Dobbin; Sunnybrook Health Sciences Centre/University of Toronto, Toronto, Ontario: Hans J. Kreder, David J. G. Stephen, Richard Jenkinson, Monica Kunz, and Ria Cagaanan; St. Michael's Hospital/University of Toronto, Toronto, Ontario: Emil H. Schemitsch, Michael D. McKee, Jeremy A. Hall, Milena R. Vicente, Lisa M. Wild, and Ryan M. Khan; Royal Columbian Hospital, New Westminster, British Columbia: Robert McCormack, Farhad Moola, Bertrand Perey, Trevor Stone, Darius Viskontas, Mike Lemke, Dory Boyer, Mauri Zomar, and Karyn Moon; McGill University Health Centre, Montreal, Quebec: Rudy Reindl, Edward Harvey, Greg Berry, Max Talbot, and Fiona Houghton; Hôpital du Sacré-Coeur de Montréal, Montreal, Quebec: Stéphane Leduc, G. Yves Laflamme, Dominique Rouleau, Sylvain Gagnon, Michel Malo, Benoit Benoit, Pierre Ranger, Julio Fernandes, Pierre Beaumont, Marie-France Poirier, and Julie Fournier; Vancouver General Hospital, Vancouver, British Columbia: Peter J. O'Brien, Piotr A. Blachut, Henry M. Broekhuyse, Pierre Guy, Kelly Lefaivre, Raman Johal, and Josephine Mutepfa; London Health Sciences Centre, London, Ontario: Abdel Lawendy, David Sanders, and Christina Tieszer; Health Sciences Centre Winnipeg, Winnipeg, Manitoba: Ted Tufescu, Brad Pilkey, Chris Graham, Laurie Barron, Jamie Dubberly, and Nigar Sultana; Toronto Western Hospital, Toronto, Ontario: Khalid Syed and Tania Azad; Foothills Medical Centre, Calgary, Alberta: Paul Duffy, Richard Buckley, Robert Korley, Shannon Puloski, Kelly Johnston, James Powell, and Kimberly Carcary.

Australia: The Alfred, Melbourne: Susan Liew, Matthias Russ, and Adam Dowrick; John Hunter Hospital and Hunter New England Area Health Service, Newcatle, NSW: Zsolt Balogh, Julie Evans, and Kate King.

Norway: Ulleval University Hospital, Oslo: Frede Frihagen, Lars Nordsletten, Jan Erik

Madsen, Gunnar Flugsrud, Anders Walloe, Geir Andreassen, and John Clarke-Jenssen.

India: Sancheti Institute for Orthopaedics & Rehabilitation: Parag Sancheti, Chetan Pradhan, Chetan Puram, Atul Patil, and Steve Rocha.

China: West China Hospital, Sichuan University: Tianfu Yang, and Yang Liu.
